# Assessment of Real-Life Outcomes in Schizophrenia Patients according to Compliance

**DOI:** 10.1155/2020/5848601

**Published:** 2020-08-31

**Authors:** Zaina P. Qureshi, Rezaul Khandker, Jason Shepherd, Salome Samant, Farid Chekani, Hollie M. L. Bailey

**Affiliations:** ^1^Merck & Co., Inc., Kenilworth, NJ, USA; ^2^Adelphi Real World, Bollington, UK

## Abstract

**Objective:**

To describe and compare demographics, outcomes and comorbidities in schizophrenia patients by treatment compliance.

**Methods:**

This was a cross-sectional survey of hospital- or office-based psychiatrists who saw ≥6 schizophrenia patients per week and were responsible for treatment decisions. Recruited physicians completed a patient record form (PRF) for their first 10 consulted schizophrenia patients aged ≥18. These patients voluntarily completed a patient self-completion form (PSC). Compliance was measured by subjective physician assessment. Drivers of and outcomes associated with compliance were identified by regression analyses.

**Results:**

A total of 150 physicians completed PRFs for 1489 patients (706 sometimes compliant (SC), 636 always compliant (AC)). A total of 680 patients completed a PSC (327 SC, 295 AC). AC patients were less likely to be male (52.2% vs. 58.6%; *P* = 0.021) and unemployed (odds ratio (OR) 0.91, 95% confidence interval (CI) 0.82–1.00; *P* < 0.001) or to have had a treatment regimen change (OR 0.56, 95% CI 0.40–0.80; *P* = 0.001) than SC patients. AC patients were less likely to have had more comorbidities (OR 0.91, 95% CI 0.82–1.00; *P* = 0.045) and hospitalizations in the past 12 months (OR 0.59, 95% CI 0.43–0.80; *P* = 0.001) than SC patients. Overall, AC patients had better clinical and humanistic outcomes. Weight gain was a common side effect for all patients; SC patients with weight gain had poorer outcomes than those without weight gain.

**Conclusion:**

Schizophrenia patients that were SC experienced poorer clinical outcomes and quality of life. Weight gain may exacerbate these poorer outcomes.

## 1. Introduction

Schizophrenia is the most common psychotic disorder [1], with approximately 7 in 1000 patients developing the disease in their lifetime [2]. Schizophrenia affects more than 23 million people worldwide [3]; approximately 2.6 million adults in the US have the disease [4]. Although absolute prevalence is low, the disease is accompanied by substantial health, social and economic burdens [5] and people with schizophrenia are two to three times more likely to die early than the general population [3]. A systematic review revealed that the total costs (direct medical, nonmedical and indirect costs) of schizophrenia across various countries ranged from US $94 million (Puerto Rico) to US $102 billion (US) [5].

The clinical profile of schizophrenia is characterized by a range of symptoms, classified as positive (psychotic symptoms including delusions and hallucinations) [1], negative (reduced emotional expression and avolition) [1] and cognitive (including disorganized speech, thought, or attention) [6–8]. Prompt initiation of pharmacotherapy after diagnosis is recommended by multiple guidelines [1]. The first-line pharmacotherapies for schizophrenia are antipsychotic agents, including first-generation (typical) and second-generation (atypical) agents. While first- and second-generation antipsychotics demonstrate equivalent effectiveness [9, 10], second-generation antipsychotics (with the exception of clozapine) are recommended as first-line pharmacotherapy [7, 11] due to their association with a lower likelihood of extrapyramidal symptoms [12]. However, second-generation antipsychotics are associated with metabolic side effects, including weight gain, hyperlipidemia, and diabetes mellitus [13], and cardiovascular side effects, such as orthostatic hypotension and reflex tachycardia [14].

Given their side effect profile, antipsychotic compliance may be a significant challenge for schizophrenia patients. A post hoc pooled analysis of four randomized double-blind clinical trials of atypical antipsychotics revealed that most patients discontinued treatment at an early stage, with poor tolerability as the second most common reason given after poor response [15]. A double-blind study comparing perphenazine with olanzapine, quetiapine, risperidone or ziprasidone revealed that up to 74% of patients discontinued medication within 18 months due to efficacy, tolerability or other reasons [16]. Other factors that influence compliance include disease severity, external environment [17] or characteristics of schizophrenia itself [18]. Poor compliance may have an adverse effect on the disease course, resulting in relapse, rehospitalization, increased time to remission and attempted suicide [19]. Improvement in positive symptoms, hostility and depressive symptoms is the best predictor of compliance [20].

Due to the importance of compliance in potentially reducing the morbidity, mortality and economic burden associated with schizophrenia, we used data from a large, multinational, cross-sectional survey of psychiatrists and their consulting patients to describe and compare the demographics, outcomes and comorbidities among schizophrenia patients according to medication compliance. These study results may facilitate the development of strategies to improve medication compliance and offer guidance on how to address the unmet needs of schizophrenia patients.

## 2. Methods

### 2.1. Study Background

Data were extracted from the Adelphi Schizophrenia Disease Specific Programme (DSP), conducted in the United States between January and May 2014. DSPs are large, cross-sectional, point-in-time surveys conducted in clinical practice that describe current disease management, disease burden impact and associated treatment patterns (both clinical and physician-perceived) in real-world clinical practice.

### 2.2. Eligible Physicians and Patients

Eligible physicians were office- or hospital-based psychiatrists who had been practicing for >2 years and <40 years at the time of study, consulted ≥6 schizophrenia patients per week and were personally responsible for their treatment decisions. Eligible patients were aged ≥18 years, had a diagnosis of schizophrenia and were not currently participating in a clinical trial.

### 2.3. Data Collection

Once recruited, physicians completed a patient record form (PRF) for the first 10 consecutively consulting patients. PRFs contain questions on patient demographics, diagnosis, management, clinical status, concomitant conditions, current treatment and treatment history.

Medication compliance was assessed via a one-time subjective physician assessment. Compliance groups were derived using the following responses: “always compliant” = always compliant (AC), “sometimes compliant” or “often compliant” = sometimes compliant (SC), and “not at all compliant” and “rarely compliant” = noncompliant. Noncompliant patients were excluded due to insufficient numbers for statistical comparison and because we chose to examine only patients with some level of compliance.

Where response to treatment was analyzed, patients were classified as responders or inadequate responders using the physician-reported Clinical Global Impression (CGI) scale on the current treatment [21]. With regard to response to treatment, patients rated as "much improved" or “very much improved” were classified as responders and patients rated as “minimally improved” were inadequate responders.

Physicians also assessed the severity of illness using the CGI scale [21]. Patients rated as “normal, not at all ill,”, “borderline mentally ill,” or “mildly ill” were considered to have mild illness severity. Patients rated as “moderately ill” or “markedly ill” were considered to have moderate illness severity, while those rated as “severely ill” or “among the most extremely ill patients” were considered to have severe illness severity.

Each patient was invited to complete a patient-reported form (PSC), containing questions on demographics, current condition and quality of life. Validated instruments in the PSC included self-rated health as assessed by the EQ-5D visual analogue scale (VAS) [22, 23], rated from 0 (worst imaginable health state) to 100 (best imaginable health state); overall life satisfaction assessed by the Quality of Life Enjoyment and Satisfaction Questionnaire (Q-LES-Q) [24], rated from 1 (very poor) to 5 (very good); and impairment assessed by Work Productivity and Activity Impairment (WPAI) [25], where higher scores indicate greater impairment. PSCs were completed by the patient independently of the physician immediately after consultation and were returned in a sealed envelope to ensure confidentiality.

### 2.4. Ethics

The DSP methodology has been previously published and validated [26–28]. Physicians provided consent to participate and provide patient information when screened for the study, with patients providing consent when completing a PSC questionnaire. Neither patients nor physicians could be identified; all data were aggregated and deidentified before receipt. Data collection was performed in accordance with the European Pharmaceutical Marketing Research Association guidelines [29]; therefore, ethics committee approval was not required. The survey was performed in accordance with the relevant legislation at the time of collection, including the US Health Insurance Portability and Accountability Act of 1996 [30] and Health Information Technology for Economic and Clinical Health Act [31].

### 2.5. Statistical Methods

All analyses were performed on Stata 15.1 (StataCorp. 2017. Stata Statistical Software: Release 15. College Station, TX: StataCorp LLC). Descriptive statistics were numeric (reported as counts and means with standard deviation) or categorical (counts and percentage of patients falling into each response). Bivariate statistical tests used to compare outcomes between groups included *t*-tests or analysis of variance for numeric variables, nonparametric Mann-Whitney *U* tests for ordered categorical variables, and Fisher's exact or chi-squared tests for nonordered categorical variables.

Regression analyses were used to determine the effect associated with being a responder, with compliance defined as the dependent variable. Regression analyses were performed independently for each outcome, after adjusting for covariates including age, gender, BMI, severity of disease, and number of comorbid conditions. The regression type was dependent on the outcome being modelled: negative binomial for count outcomes, logistic for binary outcomes, and linear regression for other continuous outcomes.

## 3. Results

### 3.1. Physicians and Patients

A total of 150 physicians completed PRFs for 1489 schizophrenia patients. Of these, 636 (42.3%) and 706 (47.4%) were considered AC or SC, respectively. Eighty-one (5.4%) patients were considered noncompliant; physician-reported compliance data were not provided for 66 (4.4%) patients. A total of 680 patients completed a PSC, independent of the physician assessment. Of these patients, 295 (43.4%) were AC, 327 (48.1%) were SC, and 35 (5.2%) were noncompliant, according to physician assessment. For 23 (3.4%) patients, compliance data was not provided.

### 3.2. Demographic and Clinical Characteristics

Mean age (40.8 vs. 41.7 years; *P* = 0.260) and BMI (both groups 28.9 kg/m^2^) were similar between SC and AC patients. SC patients were more likely to be male (58.6% vs. 52.2%; *P* = 0.021) and to be unemployed (64.9% vs. 41.4%; *P* < 0.001) (Table 1).

There was an association between SC and poor response; 58.2% of SC patients responded to their current treatment regimen compared with 80.1% of AC patients (*P* < 0.001). Compared with AC patients, SC patients more often had severe CGI illness severity (17.6 vs. 8.3; *P* < 0.001) and less often had mild illness severity (19.0 vs. 32.5; *P* < 0.001). A greater proportion of SC patients current symptoms in all recorded categories (all *P* < 0.05) except depression. SC patients had greater mean severity for positive (51.7 vs. 38.6; *P* < 0.001), negative (45.0 vs. 37.1; *P* < 0.001), and cognitive (35.9 vs. 27.1; *P* < 0.001) symptoms and had a higher mean number of comorbidities (2.3 vs. 2.0; *P* = 0.007). Physicians reported that a greater proportion of SC patients had a caregiver (39.4% vs. 31.9%; *P* = 0.008). A greater proportion of SC patients were hospitalized in 12 months (35.5% vs. 15.5%; *P* < 0.001); however, no difference in the mean number of hospitalizations in the past 12 months was observed (1.6 vs. 1.5; *P* = 0.597) (Table 2).

### 3.3. Drivers of Patient Compliance

Logistic regression analysis was performed to examine the potential drivers or predictors of compliance, with compliance as the dependent variable. Unemployed patients (odds ratio (OR) 0.50, 95% confidence interval (CI) 0.35, 0.71; *P* < 0.001) and those who have had a change in the treatment regimen (OR 0.56, 95% CI 0.40, 0.80; *P* = 0.001) were less likely to be AC patients when compared with SC patients. Patients with a greater number of comorbidities (OR 0.91, 95% CI 0.82, 1.00; *P* = 0.045) or a greater number of hospitalizations in the past 12 months (OR 0.59, 95% CI 0.43, 0.80; *P* = 0.001) were also less likely to be AC patients when compared with SC patients (Table 3).

### 3.4. Multivariate Regression Analyses

Multivariate regression analyses were performed to estimate the extent to which compliance explained various health outcomes, while controlling for other factors. We observed that AC patients were less likely to have positive symptoms (OR 0.62, 95% CI 0.42, 0.93; *P* < 0.022), anxiety (OR 0.66, 95% CI 0.48, 0.92; *P* = 0.012), sleep issues (OR 0.67, 95% CI 0.50, 0.90; *P* = 0.008), or other symptoms (OR 0.69, 95% CI 0.49, 0.96; *P* = 0.028). AC patients had less severe positive (coefficient (*β*) = −7.92, 95% CI -12.47, -3.37; *P* = 0.001), negative (*β* = −5.64, 95% CI -9.43, -1.85; *P* = 0.004), and cognitive (*β* = −5.36, 95% CI -8.95, -1.78; *P* = 0.004) symptoms. AC patients were less likely to have been hospitalized in the last 12 months (OR 0.36, 95% CI 0.25, 0.54; *P* < 0.001). In contrast to the bivariate analyses (Table 2), regression analyses revealed that AC patients had fewer hospitalizations in the last 12 months (incident rate ratio 0.41, 95% CI 0.29, 0.59; *P* < 0.001). AC patients were more likely to have no physician-reported side effects (OR 1.46, 95% CI 1.04, 2.04; *P* = 0.030) (Table 4).

AC patients were less likely to be unemployed (OR 0.46, 95% CI 0.34 to 0.64; *P* < 0.001). AC patients had better Q-LES-Q (*β* = 4.82, 95% CI 1.06, 8.57; *P* = 0.013) scores and were more likely to have fair to very good Q-LES-Q life satisfaction during the past week (OR 2.30, 95% CI 1.21, 4.41; *P* = 0.013) (Table 4).

### 3.5. Medication Side Effects and Compliance

Considering the importance of side effects on compliance and the fact that AC patients were more likely to have no physician-reported side effects, we explored possible associations between compliance and side effects. Greater proportions of AC patients experienced no side effects when compared with SC patients as reported by both physicians (47.5% vs. 34.9%; *P* < 0.001) and patients (40.7% vs. 31.0%; *P* = 0.033). Weight gain was within both the top five physician-reported and patient-reported side effects. For the top five physician- and patient-reported side effects, greater proportions of SC patients were reported to have the side effect in question except for weight gain. While AC patients were more likely to report weight gain than SC patients, this was not significant (18.6% vs. 15.7%; *P* = 0.459). In contrast, at the time of PRF completion, physicians reported that SC patients were more likely to currently experience weight gain. However, this was not significant (24.4% vs. 21.1%; *P* = 0.173) (Supplementary Table 1).

### 3.6. Weight Gain Is Associated with Poorer Outcomes in Sometimes Compliant Patients

As weight gain is an important side effect of antipsychotic therapy, we explored the possibility that weight gain exacerbates the already poorer outcomes observed in SC patients. Compared with SC patients without weight gain, those with weight gain were older (mean 45.7 vs. 40.5 years; *P* < 0.001), had higher BMI with the average of the patients being obese (mean 32.5 vs. 27.9 kg/m^2^; *P* < 0.001), and were more likely to be unemployed (53.7% vs. 36.3%; *P* = 0.014) (Supplementary Table 2). SC patients with weight gain had a higher number of concomitant conditions (mean 3.0 vs. 1.7; *P* < 0.001). Within the top five physician-reported concomitant conditions in SC patients, those with weight gain more often had hypertension (31.4% vs. 21.3%; *P* = 0.022), dyslipidemia (37.2% vs. 12.7%; *P* < 0.001), and obesity (43.8% vs. 9.2%; *P* < 0.001). Out of the top five physician-reported side effects in SC patients, those with weight gain more often had elevated lipid levels (24.6% vs. 1.5%; *P* < 0.001) (Supplementary Table 3).

## 4. Discussion

This survey of psychiatrists and schizophrenia patients revealed that SC patients are more often unemployed with more comorbidities, greater symptom severity, an increased likelihood of recent hospitalization, greater impairment and poorer general health and quality of life. Physician- and patient-reported weight gain was a common side effect, although the differences between AC and SC patients were not significant. SC patients with weight gain were more likely to be older, obese and unemployed, and to experience metabolic side effects when compared with those without weight gain.

Given the wide range of published compliance rates [32], variations in studied populations, and the challenges of defining and quantifying compliance [33], it was not possible to directly compare our results with other studies. However, our observations are consistent with other studies which conclude that partial medication compliance is often encountered in schizophrenia [34, 35] and is a challenging aspect of treatment driven by patient belief, poor medication effectiveness or tolerability, or regimen complexity [18, 36–38]. The importance of tolerability was emphasized in an interview study that revealed that schizophrenia patients considered medication as useful only if side effects are minimal and well controlled [39].

The poorer outcomes observed in the SC patients are consistent with previous observations. Noncompliance with antipsychotic regimens is associated with worsening of psychotic symptoms [40], poorer prognosis [41, 42], and increased costs [43] and use of healthcare resources [44]. An analysis of pharmacy data revealed that patients who acquired smaller proportions of their medication over time (and were considered partially compliant) were 2.4 times more likely to be hospitalized and have more hospital days than those considered to have good compliance. [45]

There was an association between patient compliance and treatment response. This relationship may be bidirectional, with response driving compliance as well as compliance driving response. For example, the improved outcomes observed in AC patients may explain in part their compliance, as these patients derive benefit from medication and therefore have incentive to be compliant. Conversely, reduced levels of compliance may also be driven by poor outcomes. AC patients had fewer comorbidities and were less likely to have had a change in the treatment regimen, which suggests that the medication burden or complexity (for schizophrenia, comorbidities, or both) may contribute to poorer compliance. Increasing the dosing frequency [46] or poor effectiveness against psychotic or negative symptoms [47] may be risk factors for noncompliance.

Antipsychotic-associated weight gain has been observed with both first- and second-generation antipsychotics [48–53]. While we did observe both patient- and physician-reported weight gain in both AC and SC patients, regression analyses did not reveal an increased risk of weight gain in AC patients when compared with SC patients. While weight gain in AC patients is an anticipated outcome of compliance and is therefore not surprising, it is possible that weight gain in SC patients may be due to prior compliance (AC status) that led to weight gain; this in turn may lead to reduced compliance (SC status).

Weight gain poses several challenges in the management of schizophrenia patients. Weight gain is an established side effect of antipsychotics and would be expected in compliant patients. However, weight gain would also be expected to increase the risk of mortality given the already high prevalence of metabolic syndrome and obesity in this population [54, 55]. Although the available evidence is limited [56], weight gain may also contribute to noncompliance [16, 57, 58], which would in turn also lead to poorer outcomes. We observed an association between poorer outcomes in SC patients and weight gain. Given the possible interactions between compliance, outcomes, and weight gain, it is plausible that proactive measures to maintain a healthy weight may prevent noncompliance and accordingly improve outcomes.

This study has some limitations. Willingness to complete the survey influenced physician participation and formal patient selection procedures were not used; therefore, this did not comprise a true random sample. The cross-sectional nature of this study limits the ability to define both compliance and response, which would have required a cohort/pre-postdesign to be robust. Furthermore, the subjective assessments used to define compliance and response may not be capturing the true compliance or response. Of the 1489 patients identified in this study, 81 (5.4%) were considered noncompliant according to physician assessment and were excluded due to insufficient numbers for statistical comparison. While it may be possible to estimate the size of this population through emergency or hospital settings, the proportion of nonattenders and noncompliant patients in the wider population is difficult to define and is likely to be underreported. Medication compliance is a subjective as well as a temporal measure, and it can be difficult to definitively assess the degree of compliance from a physician perspective. While the issue of compliance is a two-way assessment, physicians have access to a range of information when coming to a decision regarding the degree of compliance in addition to that reported by the patient. This may include, for example, prescriptions filled and caregiver reports, as well as historical medical records, to make an appropriate point-in-time assessment. As this is a real-world study, this is entirely consistent with how physician-reported compliance would be defined in normal clinical practice.

Although the point-in-time study design prevents any conclusions about causal relationships, identification of significant associations is possible. Patient and physician responses to the questionnaires may have been affected by recall bias, which is a common limitation of surveys. However, the data were collected at the time of each patient's consultation, and this is expected to reduce the likelihood of recall bias. Considerably fewer PSCs (680) were completed when compared with PRFs (1489); it is unclear if this introduced bias into the results. However, the overall proportion of AC and SC patients who were considered compliant by physicians was similar in the group of patients who provided PSCs (AC, 43.4%; SC, 48.1%) compared to the overall study population (AC, 43.4%; SC, 48.1%). Given the point-in-time nature of this study, it is not possible to determine which SC patients would have been AC if assessed at an earlier time and if weight gain is temporally associated with transitioning from AC to SC status. We postulate that the factors associated with SC patients would also be relevant in noncompliant patients and that the identification of these will aid in improving treatments and interventions. Nonetheless, further research is needed to assess disease impact and burden in this patient group.

Despite these limitations, analyses of real-world data can address concerns that are not explored in clinical trials. Patients included in clinical trials are only partially representative of the consulted schizophrenia population and may be more compliant than patients treated in real-world clinical practice.

## 5. Conclusion

On the basis of this study, improving medication compliance in schizophrenia patients may improve both clinical and humanistic outcomes. Therapeutic regimens with minimal side effects may enhance compliance. Appropriate weight management measures may also contribute to improved compliance.

## Figures and Tables

**Table 1:** Patient demographics.

	Overall	Sometimes compliant	Always compliant	*P* value (test^a^)
Age				
*n*	1338	703	635	
Mean (SD)	41.2 (14.7)	40.8 (14.6)	41.7 (14.8)	0.260 (TT)
Gender				
*n*	1341	705	636	
Male	745 (55.6)	413 (58.6)	332 (52.2)	0.021 (FE)
BMI (kg/m^2^)				
*n*	1167	611	556	
Mean (SD)	28.9 (6.3)	28.9 (6.3)	28.9 (6.3)	0.946 (TT)
Patient current employment				
*n*	1331	701	630	
Full-time	172 (12.9)	48 (6.8)	124 (19.7)	<0.001 (CH)
Part-time	206 (15.5)	98 (14.0)	108 (17.1)	
Homemaker	86 (6.5)	30 (4.3)	56 (8.9)	
Student	90 (6.8)	42 (6.0)	48 (7.6)	
Retired	61 (4.6)	28 (4.0)	33 (5.2)	
Unemployed	716 (53.8)	455 (64.9)	261 (41.4)	

BMI = body mass index; CH = chi-squared test; FE = Fisher's exact test; SD = standard deviation; TT = Student's *t*-test. ^a^Statistical test performed. All data reported as *n* (%) unless indicated otherwise.

**Table 2:** Patient clinical characteristics and hospitalizations.

	Overall	Sometimes compliant	Always compliant	*P* value (test^a^)
Response to the current treatment regimen				
*n*	1236	644	592	
Inadequate responder	387 (31.3)	269 (41.8)	118 (19.9)	<0.001 (FE)
Responder	849 (68.7)	375 (58.2)	474 (80.1)	
CGI overall impression of severity of illness				
*n*	1334	700	634	
Mild	339 (25.4)	133 (19.0)	206 (32.5)	<0.001 (CH)
Moderate	820 (61.5)	444 (63.4)	376 (59.3)	
Severe	175 (13.1)	123 (17.6)	52 (8.2)	
Current symptoms present				
*n*	1342	706	636	
Positive	1114 (83.0)	619 (87.7)	495 (77.8)	<0.001 (FE)
Negative	1168 (87.0)	628 (89.0)	540 (84.9)	0.028 (FE)
Cognitive impairments	956 (71.2)	539 (76.3)	417 (65.6)	<0.001 (FE)
Anxiety	918 (68.4)	515 (72.9)	403 (63.4)	<0.001 (FE)
Depression	757 (56.4)	396 (56.1)	361 (56.8)	0.826 (FE)
Sleep issues	493 (36.7)	293 (41.5)	200 (31.4)	<0.001 (FE)
Other	302 (22.5)	185 (26.2)	117 (18.4)	<0.001 (FE)
Overall severity, positive symptoms^b^				
*n*	1326	699	627	
Mean (SD)	45.5 (29.4)	51.7 (28.5)	38.6 (28.8)	<0.001 (TT)
Overall severity, negative symptoms^b^				
*n*	1328	701	627	
Mean (SD)	41.3 (24.3)	45.0 (24.6)	37.1 (23.3)	<0.001 (TT)
Overall severity, cognitive symptoms^b^				
*n*	1327	701	626	
Mean (SD)	31.8 (24.4)	35.9 (24.8)	27.1 (23.0)	<0.001 (TT)
Number of concomitant conditions				
*n*	1336	704	632	
Mean (SD)	2.1 (2.1)	2.3 (2.1)	2.0 (2.1)	0.007 (TT)
Concomitant conditions				
*n*	1336	704	632	
Anxiety	330 (24.7)	184 (26.1)	146 (23.1)	0.204 (FE)
Hypertension	344 (25.7)	194 (27.6)	150 (23.7)	0.117 (FE)
Depression	257 (19.2)	138 (19.6)	119 (18.8)	0.729 (FE)
Obesity	230 (17.2)	125 (17.8)	105 (16.6)	0.612 (FE)
Dyslipidemia	229 (17.1)	120 (17.0)	109 (17.2)	0.942 (FE)
Stress	185 (13.8)	115 (16.3)	70 (11.1)	0.006 (FE)
Insomnia	132 (9.9)	86 (12.2)	46 (7.3)	0.003 (FE)
Diabetes	136 (10.2)	73 (10.4)	63 (10.0)	0.856 (FE)
Hospitalized because of disease in the last 12 months				
*n*	1314	693	621	
Hospitalized	342 (26.0)	246 (35.5)	96 (15.5)	<0.001 (FE)
Number of hospitalizations in the last 12 months^c^				
*N*	278	203	75	
Mean (SD)	1.5 (1.0)	1.6 (0.9)	1.5 (1.2)	0.597 (TT)
Caregiver status				
*n*, physician-reported	1240	648	592	
Has caregiver	444 (35.8)	255 (39.4)	189 (31.9)	0.008 (FE)
*n*, patient-reported	605	318	287	
Has caregiver	214 (35.4)	118 (37.1)	96 (33.4)	0.351 (FE)

CGI = Clinical Global Impression; CH = chi-squared test; FE = Fisher's exact test; SD = standard deviation; TT = Student's *t*-test. ^a^Statistical test performed. ^b^Rated from 0 (not present) to 100 (severe). ^c^Only patients who were hospitalized were included. All data reported as *n* (%) unless indicated otherwise.

**Table 3:** Drivers of patient compliance.

	Odds ratio (95% CI)	*P* value
Age	1.01 (1.00, 1.02)	0.054
Gender		
Male	1 (base)	
Female	1.08 (0.83, 1.39)	0.580
BMI	1.02 (1.00, 1.04)	0.203
Employment		
Employed	1 (base)	
Unemployed	0.50 (0.35, 0.71)	<0.001
Number of comorbidities	0.91 (0.82, 1.00)	0.045
Caregiver status		
No caregiver	1 (base)	
Nonprofessional caregiver	1.06 (0.72, 1.54)	0.781
Professional caregiver	1.30 (0.77, 2.19)	0.329
Number of hospitalizations in the past 12 months	0.59 (0.43, 0.80)	0.001
Change in the treatment regimen		
No change	1 (base)	
Change	0.56 (0.40, 0.80)	0.001
CGI overall impression of illness severity		
Mild	1 (base)	
Moderate	0.90 (0.60, 1.35)	0.593
Severe	0.57 (0.32, 1.05)	0.070

Logistic regression analysis, with compliance used as the dependent variable. Results were based on 994 observations. Odds ratios are based on the patient being always compliant (always compliant = 1, sometimes compliant = 0). Abbreviations: CGI = Clinical Global Impression; CI = confidence interval.

**Table 4:** Multivariate regression analyses.

	*n* ^a^	Value^b^ (95% CI)	*P* value
Responder	1137	2.51 (1.84, 3.42)	<0.001
Symptoms			
Current symptoms present			
Positive	1153	0.62 (0.42, 0.93)	0.022
Negative	1153	0.82 (0.55, 1.25)	0.361
Cognitive impairments	1153	0.72 (0.51, 1.01)	0.060
Anxiety	1153	0.66 (0.48, 0.92)	0.012
Depression	1153	1.00 (0.73, 1.36)	0.974
Sleep issues	1153	0.67 (0.50, 0.90)	0.008
Other	1153	0.69 (0.49, 0.96)	0.028
Overall severity^c^			
Positive	1144	-7.92^d^ (-12.47, -3.37)	0.001
Negative	1144	-5.64^d^ (-9.43, -1.85)	0.004
Cognitive	1144	-5.36^d^ (-8.95, -1.78)	0.004
Hospitalizations			
Hospitalized in the last 12 months	1129	0.36 (0.25, 0.54)	<0.001
Number of hospitalizations in the last 12 months	1076	0.41^e^ (0.29, 0.59)	<0.001
Medication side effects			
Physician-reported			
None	1053	1.46 (1.04, 2.04)	0.030
Weight gain	1053	0.97 (0.67, 1.39)	0.849
Patient-reported			
None	394	1.43 (0.89, 2.28)	0.138
Weight gain	394	1.45 (0.74, 2.83)	0.282
Employment, caregiver, quality of life, and impairment			
Unemployed	1143	0.46 (0.34, 0.64)	<0.001
Has caregiver (physician-reported)	1074	0.99 (0.72, 1.36)	0.949
Has caregiver (patient-reported)	527	0.98 (0.65, 1.48)	0.936
EQ-5D health utility	444	0.03^d^ (-0.01, 0.07)	0.119
EQ-5D VAS	432	3.31^d^ (-0.71, 7.32)	0.106
Q-LES-Q score	407	4.82^d^ (1.06, 8.57)	0.013
Q-LES-Q life satisfaction/contentment during the past week^f^	441	2.30 (1.21, 4.41)	0.012
WPAI			
Percent work time missed	147	-6.27^d^ (-14.75, 2.22)	0.145
Percent impairment while working	144	-2.08^d^ (-9.56, 5.40)	0.581
Percent overall work impairment	141	-2.02^d^ (-11.06, 7.02)	0.657
Percent activity impairment	494	1.11d (-3.73, 5.95)	0.650

Each row represents a separate regression. Results are based on the patient being a responder. CI = confidence interval; EQ-5D = EuroQol 5 Dimensions; Q-LES-Q = Quality of Life Enjoyment and Satisfaction Questionnaire; VAS = visual analogue scale; WPAI = Work Productivity and Activity Impairment. ^a^Number of observations. ^b^All values are odds ratios unless otherwise indicated. Odds ratios were calculated based on the patient being always compliant (always compliant = 1, sometimes compliant = 0). ^c^Measured from 0 (not present) to 100 (severe). ^d^Coefficient (*β*). ^e^Incidence rate ratio. ^f^Patients who responded with fair, good, or very good.

## Data Availability

All data that support the findings of this study are intellectual property of Adelphi Real World. All requests should be addressed directly to Jason Shepherd at jason.shepherd@adelphigroup.com.

## Supplementary Materials

Supplementary Materials Supplementary Table 1: medication side effects and compliance. Supplementary Table 2: demographics of sometimes compliant patients by weight gain. Supplementary Table 3: clinical characteristics and hospitalizations of sometimes compliant patients by weight gain.
