# Supplementary material for: Assessment of Real-Life Outcomes in Schizophrenia Patients according to Compliance
Source: Psychiatry J. 2020 Aug 31;2020:5848601. doi: 10.1155/2020/5848601 (PMC7479455; doi:10.1155/2020/5848601)
Supplement: Supplementary Materials — Supplementary Table 1: medication side effects and compliance. Supplementary Table 2: demographics of sometimes compliant patients by weight gain. Supplementary Table 3: clinical characteristics and hospitalizations of sometimes compliant patients by weight gain. [file 5848601.f1.docx]

# **Supplementary Tables**

| **Supplementary Table 1.** Medication side effects and compliance | | | | |
| --- | --- | --- | --- | --- |
|  | Overall | Sometimes compliant | Always compliant | *P*-value (test^a^) |
| Side effects |  |  |  |  |
| n, physician-reported | 1226 | 647 | 579 |  |
| No side effects | 501 (40.9) | 226 (34.9) | 275 (47.5) | < 0.001 (FE) |
| n, patient-reported | 460 | 229 | 231 |  |
| No side effects | 165 (35.9) | 71 (31.0) | 94 (40.7) | 0.033 (FE) |
|  |  |  |  |  |
| Top 5 reported side effects |  |  |  |  |
| n, physician-reported | 1226 | 647 | 579 |  |
| Sedation | 339 (27.7) | 206 (31.8) | 133 (23.0) | < 0.001 (FE) |
| Any metabolic | 308 (25.1) | 179 (27.7) | 129 (22.3) | 0.035 (FE) |
| Weight gain | 280 (22.8) | 158 (24.4) | 122 (21.1) | 0.173 (FE) |
| Extrapyrimidal symptoms | 130 (10.6) | 90 (13.9) | 40 (6.9) | < 0.001 (FE) |
| Anticholinergic effects | 102 (8.3) | 68 (10.5) | 34 (5.9) | 0.004 (FE) |
|  |  |  |  |  |
| n, patient-reported | 460 | 229 | 231 |  |
| Tiredness/lethargy | 130 (28.3) | 70 (30.6) | 60 (26.0) | 0.301 (FE) |
| Sedation/drowsiness | 119 (25.9) | 62 (27.1) | 57 (24.7) | 0.595 (FE) |
| Poor concentration | 94 (20.4) | 57 (24.9) | 37 (16.0) | 0.021 (FE) |
| Weight gain | 79 (17.2) | 36 (15.7) | 43 (18.6) | 0.459 (FE) |
| Confusion | 61 (13.3) | 43 (18.8) | 18 (7.8) | < 0.001 (FE) |
| FE = Fisher’s exact test.  ^a^Indicates statistical test performed.  All data reported as n (%) unless indicated otherwise. | | | | |

| **Supplementary Table 2**. Demographics of sometimes compliant patients by weight gain | | | | |
| --- | --- | --- | --- | --- |
|  | Overall | No weight gain | Weight gain | *P*-value (test^a^) |
| Age |  |  |  |  |
| n | 578 | 456 | 122 |  |
| Mean (SD) | 41.6 (14.8) | 40.5 (14.9) | 45.7 (13.6) | <0.001 (TT) |
| Gender |  |  |  |  |
| n | 579 | 457 | 122 |  |
| Male | 303 (52.3) | 235 (51.4) | 68 (55.7) | 0.416 (FE) |
| BMI, kg/m^2^ |  |  |  |  |
| n | 506 | 405 | 101 |  |
| Mean (SD) | 28.8 (6.2) | 27.9 (5.5) | 32.5 (7.2) | <0.001 (TT) |
| Patient current employment |  |  |  |  |
| n | 575 | 454 | 121 |  |
| Full-time | 118 (20.5) | 103 (22.7) | 15 (12.4) | 0.014 (CH) |
| Part-time | 102 (17.7) | 84 (18.5) | 18 (14.9) |  |
| Homemaker | 51 (8.9) | 42 (9.3) | 9 (7.4) |  |
| Student | 45 (7.8) | 38 (8.4) | 7 (5.8) |  |
| Retired | 29 (5.0) | 22 (4.8) | 7 (5.8) |  |
| Unemployed | 230 (40.0) | 165 (36.3) | 65 (53.7) |  |
| BMI, body mass index; CH, Chi-squared test; FE, Fisher’s exact test; SD, standard deviation; TT, student’s T-test  ^a^Indicates statistical test performed.  All data reported as n (%) unless indicated otherwise. | | | | |

| **Supplementary Table 3**. Clinical characteristics and hospitalizations of sometimes compliant patients by weight gain | | | | |
| --- | --- | --- | --- | --- |
|  | Overall | No weight gain | Weight gain | *P*-value (test^a^) |
| Response to current treatment regimen |  |  |  |  |
| n | 540 | 421 | 119 |  |
| Inadequate responder | 108 (20.0) | 92 (21.9) | 16 (13.4) | 0.051 (FE) |
| Responder | 432 (80.0) | 329 (78.1) | 103 (86.6) |  |
| Current symptoms present |  |  |  |  |
| n | 579 | 457 | 122 |  |
| Positive | 449 (77.5) | 354 (77.5) | 95 (77.9) | 1.000 (FE) |
| Negative | 489 (84.5) | 381 (83.4) | 108 (88.5) | 0.205 (FE) |
| Cognitive impairments | 379 (65.5) | 297 (65.0) | 82 (67.2) | 0.670 (FE) |
| Anxiety | 367 (63.4) | 292 (63.9) | 75 (61.5) | 0.672 (FE) |
| Depression | 330 (57.0) | 262 (57.3) | 68 (55.7) | 0.759 (FE) |
| Sleep issues | 180 (31.1) | 142 (31.1) | 38 (31.1) | 1.000 (FE) |
| Other | 109 (18.8) | 68 (14.9) | 41 (33.6) | <0.001 (FE) |
| Overall severity, positive symptoms^b^ |  |  |  |  |
| n | 571 | 449 | 122 |  |
| Mean (SD) | 38.4 (29.1) | 37.2 (28.3) | 42.7 (31.3) | 0.059 (TT) |
| Overall severity, negative symptoms^b^ |  |  |  |  |
| n | 570 | 448 | 122 |  |
| Mean (SD) | 36.4 (23.4) | 36.3 (23.9) | 36.7 (21.6) | 0.873 (TT) |
| Overall severity, cognitive symptoms^b^ |  |  |  |  |
| n | 569 | 447 | 122 |  |
| Mean (SD) | 26.6 (23.0) | 27.2 (23.0) | 24.6 (23.2) | 0.281 (TT) |
| Number of concomitant conditions |  |  |  |  |
| n | 576 | 455 | 121 |  |
| Mean (SD) | 1.9 (2.0) | 1.7 (1.9) | 3.0 (2.2) | <0.001 (TT) |
| Top 5 physician-reported concomitant conditions |  |  |  |  |
| n | 576 | 455 | 121 |  |
| Hypertension | 135 (23.4) | 97 (21.3) | 38 (31.4) | 0.022 (FE) |
| Anxiety | 127 (22.0) | 97 (21.3) | 30 (24.8) | 0.459 (FE) |
| Depression | 109 (18.9) | 81 (17.8) | 28 (23.1) | 0.193 (FE) |
| Dyslipidemia | 103 (17.9) | 58 (12.7) | 45 (37.2) | <0.001 (FE) |
| Obesity | 95 (16.5) | 42 (9.2) | 53 (43.8) | <0.001 (FE) |
| Top 5 physician-reported side effects |  |  |  |  |
| n | 579 | 457 | 122 |  |
| Sedation | 133 (23.0) | 97 (21.2) | 36 (29.5) | 0.069 (FE) |
| Any metabolic | 129 (22.3) | 7 (1.5) | 122 (100.0) | <0.001 (FE) |
| Weight gain | 122 (21.1) | 0 (0.0) | 122 (100.0) | <0.001 (FE) |
| Extrapyrimidal symptoms | 40 (6.9) | 30 (6.6) | 10 (8.2) | 0.547 (FE) |
| Elevated lipid levels | 37 (6.4) | 7 (1.5) | 30 (24.6) | <0.001 (FE) |
| Hospitalized because of disease in last 12 months |  |  |  |  |
| n | 565 | 447 | 118 |  |
| Hospitalized | 85 (15.0) | 69 (15.4) | 16 (13.6) | 0.667 (FE) |
| Number of hospitalizations in last 12 months^c^ |  |  |  |  |
| n | 66 | 53 | 13 |  |
| Mean (SD) | 1.5 (1.2) | 1.5 (1.3) | 1.4 (0.7) | 0.673 (TT) |
| Caregiver status |  |  |  |  |
| n, physician-reported | 540 | 424 | 116 |  |
| Has caregiver | 174 (32.2) | 132 (31.1) | 42 (36.2) | 0.314 (FE) |
| n, patient-reported | 260 | 191 | 69 |  |
| Has caregiver | 85 (32.7) | 60 (31.4) | 25 (36.2) | 0.459 (FE) |
| CH, Chi-squared test; FE, Fisher’s exact test; SD, standard deviation; TT, student’s T-test  ^a^Indicates statistical test performed.  ^b^Rated from 0 (not present) to 100 (severe).  ^c^Only patients who were hospitalized included.  All data reported as n (%) unless indicated otherwise. | | | | |
